# The Role of Cholesterol during the Ovarian Maturation and Lipid Metabolism of Female Chinese Mitten Crab (*Eriocheir sinensis*)

**DOI:** 10.1155/2024/9933600

**Published:** 2024-01-31

**Authors:** Huixing Guo, Haokun Hua, Jianfeng Wang, Wei Qiang, Xiaoe Xiang, Wenbin Liu, Guangzhen Jiang

**Affiliations:** Key Laboratory of Aquatic Nutrition and Feed Science of Jiangsu Province, College of Animal Science and Technology, Nanjing Agricultural University, No.1 Weigang Road, Nanjing 210095, China

## Abstract

In previous study, we found that the cholesterol requirement of *Eriocheir sinensis* was 0.27%, to further investigate the effects of cholesterol on health status, ovarian maturation, and lipid metabolism of female *Eriocheir sinensis* broodstock. Two diets containing 0% and 0.25% (actually 0.05% and 0.27%) cholesterol were fed to the female crabs (average weight: 49.21 ± 0.11 g) for 4 months and sampled once a month. The results showed that the body weight (BW), survival rate (SR), meat yield (MY), condition factor (CF), hepatosomatic index (HSI), and gonadosomatic index (GSI) of *Eriocheir sinensis* were significantly affected by treatment time and compared with the cholesterol deficient group, supplementing cholesterol significantly increased BW, HSI, and GSI (*P* < 0.05). In addition, long-term lack of cholesterol will lead to a significant decrease in the activity of ACP, AKP, and SOD and a significant increase in the content of MDA. The histological results showed that cholesterol significantly increased the volume of oocytes (*P* < 0.05). Further studies found that 0.27% cholesterol significantly increased the transcription levels of *vtg* and *vgr* in hepatopancreas and ovaries, which may be the main reason for the increase of oocyte size (*P* < 0.05). When fed with 0.27% cholesterol diet, the contents of nutrients in hepatopancreas and ovaries increased significantly, especially lipids and cholesterol (*P* < 0.05). Through the analysis of mRNA expression level of genes related to lipid metabolism, it was found that cholesterol enhanced the transcription level of genes related to lipid synthesis and transport in hepatopancreas, thereby promoting the accumulation of lipid in the organism. Furthermore, compared with control group, the levels of juvenile hormone (JH), 17*β*-estradiol (E_2_), methyl farnesoate (MF), and ecdysone in the organism were significantly increased after feeding a diet with 0.27% cholesterol (*P* < 0.05). In summary, supplementing an appropriate amount of cholesterol in the diet can improve the growth performance of *Eriocheir sinensis* broodstock, enhance the body's antioxidant and immune system, and promote the accumulation of nutrients in the ovaries, thereby promoting ovarian maturation.

## 1. Introduction

*Eriocheir sinensis* is one of the most important economic aquatic animals in China. In recent years, with the rapid development of aquaculture, the output of *Eriocheir sinensis* has continued to increase, and by 2022, it has exceeded 800,000 t [1–3]. However, due to long-term fishing, the destruction of natural spawning grounds, the rapid expansion of the market, and the natural germplasm resources of *Eriocheir sinensis* are gradually decreasing [4, 5]. High-quality fry crab is the key to the sustainable development of *Eriocheir sinensis* cultural industry [6, 7]. Therefore, it is necessary to artificially cultivate broodstock and breed fry crab to meet the market demand. Previous studies have shown that the gonadal maturity is closely related to the quality of fry crab [8]. Currently, the cultivation of *Eriocheir sinensis* mainly relies on feeding trash fish, but there are many problems in this feeding mode. For example, the supply of trash is unstable, the price fluctuates greatly, and the environmental pollution is serious [8, 9]. Therefore, it is of great significance for the sustainable development of *Eriocheir sinensis* breeding industry to formulate compound feed with more comprehensive nutrition based on the nutritional characteristics of broodstock so as to improve the quality of fry crab.

The broodstock nutrition of shrimp and crab has always been a weak point in the study of aquatic nutrition. Currently, research on the nutrition of shrimp and crab is mostly focused on the juvenile stage [10–14]. However, shrimps and crabs have different nutrient requirements at different growth stages, especially during the ovarian development stage, the broodstock needs to accumulate a large amount of nutrients to meet the requirements of ovarian development [9, 15, 16]. For *Eriocheir sinensis*, under natural conditions, its gonads begin to develop in the late summer of the following year, mature in late autumn, and then begin to mate and hatch eggs. During this period, the body needs to utilize a large amount of substances such as lipid, protein, and vitamins to synthesize vitellogenin (VTG), which is the main energy source for embryos and early larvae [1, 17]. Shrimps completely rely on VTG nutrition for survival and development within 48 hr of hatching [18]. Therefore, promoting the synthesis of VTG through nutrients is an important way to improve broodstock quality.

Cholesterol, as a lipid substance, plays an important physiological function in the body. First of all, cholesterol, as a key component of cell membrane, is crucial to maintain the immune function of the body. Previous research show that supplementing cholesterol in a casein-based purified diets can enhance the nonspecific immune ability of *Portunus trituberculatus* [19]. Second, lipoprotein is a mediator of lipid transport in the hemolymph of crustaceans, and one of its main components is cholesterol. Therefore, cholesterol is of great significance for lipid metabolism in crustaceans. According to previous studies, cholesterol may promote lipid accumulation in the body by promoting the expression of lipid synthesis-related genes (*srebp-1*, *fas*) while inhibiting the expression of *β*-oxidation-related gene (*cpt1*) at the transcriptional level, the transport of lipid in *Eriocheir sinensis* organs mainly depends on the assistance of fatty acid transport protein (FATP; *fatp4*, *fatp6*) and fatty acid-binding protein (FABP; *fabp3*, *fabp9*, *fabp10*). FATP is involved in the transport of fatty acid between cells, while FABP is responsible for intracellular transport [9, 11]. Moreover cholesterol is the precursor for the synthesis of various sterol hormones, which is essential for growth and development [20–22]. Therefore, cholesterol is of great significance to the growth and reproduction of aquatic animals, especially for crustaceans, they lack the ability to synthesize cholesterol, and obtaining cholesterol from exogenous foods is the only way for them [23]. At present, it has been reported that cholesterol can promote the reproduction of some crustaceans. Liang et al. [8] found that adding cholesterol can promote the ovarian maturation of *Litopenaeus vannamei*. Wu et al. [24] reported that the dietary cholesterol level is closely related to the number of eggs and the quality of larvae of *Portunus trituberculatus*. In recent years, there have also been some studies on the effects of cholesterol on *Eriocheir sinensis*, while the sutdy on cholesterol nutrition of *Eriocheir sinensis* is mainly focused on the larval stage. There are few studies to investigate the effects of cholesterol on adult crab. However, for *Eriocheir sinensis*, ovarian maturity can determine its value. Therefore, the effect of cholesterol on ovarian development is worthy of attention. For example, our previous study confirmed that cholesterol has a certain promoting effect on the ovary maturation of *Eriocheir sinensis* [15]. However, there is currently no long-term research on the impact of cholesterol on the ovarian development and health status of *Eriocheir sinensis*. Therefore, this study through a dynamic sampling experiment to investigate the role of cholesterol in the whole process of broodstock cultivation, so as to enrich the basic research on broodstock nutrition of *Eriocheir sinensis*, and promote the preparation of comprehensive formula feed and the sustainable development of the crab breeding industry.

## 2. Materials and Methods

All operations involved in this experiment were authorized by the Animal Welfare Committee of Nanjing Agricultural University (Nanjing, China) (No. SYXK (Su) 2011–0036).

### 2.1. Experimental Diets

This study is a continuation of our previous experiment. Previously, we evaluated the effects of 0%, 0.1%, 0.2%, 0.4%, 0.8%, and 1.6% cholesterol on the growth performance of *Eriocheir sinensis* and concluded that the growth requirement of *Eriocheir sinensis* for cholesterol was 0.27% [9], so a group with 0.27% cholesterol was set in this experiment. Two isolipidic and isonitrogenous diets (8% lipid and 36% protein) were prepared to add 0% and 0.25% cholesterol (actually 0.05% and 0.27%, record separately as Control and CHO 0.25) (Table 1). The production process of experiment diets refers to the previous study of our laboratory [9]. All the ingredients were smashed and sifted through a 60-mesh percolator. Then, all the raw materials were weighed and mixed thoroughly with oil sources. Thereafter, about 30% deionized water was added for further homogenization. Subsequently, the homogeneous mixture was squeezed into a 2.5 mm diameter using a single-screw meat grinder extruder. Finally, the pellets were air dried (27°C) for 24 hr and stored at −20°C in a vacuum plastic bags.

### 2.2. Experimental Animals and Feeding Trial

This experiment was carried out in Aquatic Teaching Base of Nanjing Agricultural University (Nanjing, Jiangsu, China). The experimental crabs were provided by Huahai Seed Co. Ltd., in Nanjing (Jiangsu China). Prior to the feeding trial, all crabs were put into the square pool for a week to adapt to the experimental environment. After the adaptation period, a total of 800 healthy female crabs (average weight, 49.21 ± 0.11 g) with intact appendages were randomly allotted into eight square pools, 100 crabs in each square pool, and each treatment containing four square pools (5 × 2 × 1 m, L × W × H). Eighty plastic pipes were placed in each square pool as shelters for crabs to hide. All crabs were hand-fed with corresponding diets with 4% body weight once daily (17:30) for 4 months. The experimental period is 4 months (from June to October). During this period, the feces were scavenged in the morning every day, and 30% volume of fresh water was added. The water quality was monitored daily to ensure that the dissolved oxygen >5 mg/L, the ammonia nitrogen <0.05 mg/L, the pH between 7.4 and 8.4, and the water temperature between 23°C and 28°C.

### 2.3. Sample Collection

All crabs were first sampled on June 1st (June) and then sampled once a month (July, August, September, October). Before sampling, all crabs were starved for 24 hr. Then the crabs in each replicate were weighed and counted to calculate growth performance-related parameters. Ten individuals were randomly selected from each square pool and quickly anesthetized with ice, then the length and width of crabs were measured with vernier calipers. Thereafter, extracting the hemolymph from the second appendage with a 1 mL syringe, centrifuging the hemolymph at 4°C, 4,500 rpm for 15 min, then remove the supernatant into a 0.2 mL centrifuge tube and store at −20°C for biochemical indicators. After that, all crabs were dissected, the muscle, hepatopancreas, and ovary were quickly taken out and weighted. Afterward, the hepatopancreas and part of the ovaries were stored in freezing tubes at −80°C, and the remaining ovary was stored in 4% paraformaldehyde for subsequent histological analysis.

### 2.4. Proximate Composition Analysis

The proximate compositions of diets were analyzed according to standard methods [25]. The content of moisture was measured by drying in an oven at 105°C to constant weight. The crude protein was determined using the Kjeldahl method (FOSS KT260, Swiss). The content of ether extract was determined with anhydrous ether (40–60°C) using the Soxtec system. The ash content was determined by burning in muffle furnace at 550°C to constant weight. The content of cholesterol in the diet was detected by high-performance liquid chromatography (HPLC; Agilent ZORBAX Eclipse Plus, column C18 5 *μ*m 4 : 6 × 150 mm). The specific process refers to the previous research of our laboratory [9]. The detection method of total lipid in hepatopancreas and ovary is as follows: 1 g tissue sample was taken, add 4 mL extraction solution (*ν* chloroform/*ν* methanol = 2/1), homogenate for 4°C for 5 min, add 0.88% KCl solution, fully mix for 30 min, and then dry with nitrogen blower to get total lipid. Determination of crude protein in the hepatopancreas and ovary was performed as in the diet.

### 2.5. Biochemical Analysis

About 0.2 g of hepatopancreas and ovary were taken, respectively, and then added precooled physiological saline in the ratio of 1 : 9 for homogenization. Subsequently, centrifuged at 3,000 rpm for 5 min, and the supernatant was taken for the determination of biochemical indicators, the methods are as follows: cholesterol oxidase–peroxidase (COD-PAP) single reagent colorimetry was used to determine total cholesterol (TC) content; *α*-ketoglutaric acid was used to determine aspartate aminotransferase (AST) and alanine transaminase (ALT) activity, respectively, AST can cause *α*-ketoglutaric acid and aspartic acid to generate oxalacetic acid, ALT acts on alanine and *α*-ketoglutaric acid to generate pyruvate; the activity of alkaline phosphatase (AKP) and acid phosphatase (ACP) was determined by disodium benzene phosphate; and the determination of the glutathione peroxidase (GPx) refers to the description of Lygren et al. [26], using H_2_O_2_ to determine; the activity of superoxide dismutase (SOD) was determined by the WST-1 method; the content of malondialdehyde (MDA) was determined using the red product generated by the condensation of MDA and thiobarbituric acid (TBA), the standards and commercial kits used in the above experiment were provided by Jiancheng Bioengineering Co. (Nanjing, China). The contents of vitellogenin (VTG), estradiol (E_2_), juvenile hormone (JH), and methyl farnesoate (MF) were detected by ELISA kits, where the antibodies to VTG were from crabs, while the antibodies to E_2_, JH, and MF were from insects, and were provided by Enzyme Bioengineering Co. (Shanghai, China). The specific operations were strictly in accordance with the instructions.

### 2.6. Ovarian Histology Analysis

The fixed ovary was dehydrated in a gradient alcohol and subsequently placed in xylene to replace the alcohol. The dehydrated tissues were embedded in paraffin, then the paraffin was cut into 6 *µ*m slices with a microtome (LeicaRM2016, Berlin, Germany), and applied to the slide. Subsequently, the sections were dewaxed and rehydrated so that the dye could enter the tissue. After rehydration, hematoxylin–eosin (H&E) staining was performed. Then the film was transparent with xylene, sealed with resin, observed under a microscope (Nikon Eclipse 80i, Tokyo, Japan), and photographed by a digital camera (Nikon DS-U2, Tokyo, Japan). Thereafter, the long diameter of an oocyte (LO) and short diameter of an oocyte (SO) were measured using Image-Pro Plus 6.0 (America media cybernetics). Forty oocytes from four replicate crabs and eight slides were analyzed. Calculate the volume of oocyte (VO) based on the above parameters, and the specific calculation formula are as follows [15]:

(1) $$\begin{array}{c} \text{VO}=0.523\times W_{\text{o}}^{2}\times L_{\text{o}}, \end{array}$$

where *L*_o_ = the maximum length of oocyte and *W*_o_ = the maximum width of oocyte.

### 2.7. Genes Expression

The total RNA in hepatopancreas and ovary were extracted by Trizol (Accurate, Hunan, China). The concentration of RNA was measured by a microspectrophotometer (NanoDrop 2000, USA), and the purity of RNA was assessed by OD260/OD280. Then 1 *µ*g of RNA was rapidly reverse-transcribed to cDNA by the ExScript RT-qPCR Kit (Takara, Dalian, China) for subsequent real-time quantitative PCR (RT-qPCR) analysis. The RT-qPCR was conducted in a real-time detector (Bio-rad, Richmond) and the reaction system as follows: 10 *µ*L SYBR regent (Takara, Dalian, China), 2 *µ*L cDNA template, forward primers and reverse primers each 0.4 *µ*L and 7.2 *µ*L DEPC-treated water. The specific amplification program and the melting curve refer to the Guo et al. [9]. The *β*-actin as internal reference gene (genes of crab sampled in June as the calibrator), and the expression of *vtg*, *vgr*, *srebp-1*, *fas*, *cpt1*, *mttp*, *fatp4*, *fatp6*, *fabp3*, *fabp9*, *and fabp10* were calculated using the 2^−*ΔΔCT*^ method [27]. The primer sequences involved in this experiment as shown in Table 2.

### 2.8. The Calculations of Growth Parameters

The relevant parameters are calculated according to the following formulas:

(2) $$\begin{array}{c} \text{BW}\left(g\right)=\frac{\text{Total weight of crabs}}{\text{The number of crabs}}, \end{array}$$

(3) $$\begin{array}{c} \text{SR}\left(\%\right)=\frac{\text{The number of survival crabs}}{\text{Total number of crabs}}, \end{array}$$

(4) $$\begin{array}{c} \text{MY}\left(\%\right)=\frac{\text{Total weight of muscle}}{\text{The body weight of crab}}, \end{array}$$

(5) $$\begin{array}{c} \text{CF}\left(\%\right)=100\%\times \frac{\text{The body weight of crab}}{\text{The body length of crab}{\left(\text{cm}\right)}^{3}}, \end{array}$$

(6) $$\begin{array}{c} \text{HSI}\left(\%\right)=100\%\times \frac{\text{The weight of hepatopancreas}}{\text{The body weight of crab}}, \end{array}$$

(7) $$\begin{array}{c} \text{GSI}\left(\%\right)=100\%\times \frac{\text{The weight of ovary}}{\text{The body weight of crab}}, \end{array}$$

where BW is body weight, SR is survival rate, MY is meat yield, CF is condition factor, HSI is hepatosomatic index, and GSI is gonadosomatic index.

### 2.9. Statistical Analysis

All data in this experiment contain four replicates and are presented as means with standard error of means (SEM). Before analysis, normality and homoscedasticity were checked by Kolmogorov–Smirnov test and Levene's tests (SPSS 23.0, USA). Then one-way analysis of variance was conducted to evaluate the effect of different treatment time in the same cholesterol, and Duncan's multiple comparison test was performed to analyze the significant difference (*a*, *b*, *c*, *A*, *B*, *C*). In the same treating time, the independent samples test was used to analyze the effects of different cholesterol levels on the results. The asterisk “ ^*∗*^” represents significance difference. The significant level is *P* < 0.05.

## 3. Results

### 3.1. Growth Performance

**Figure 1:**
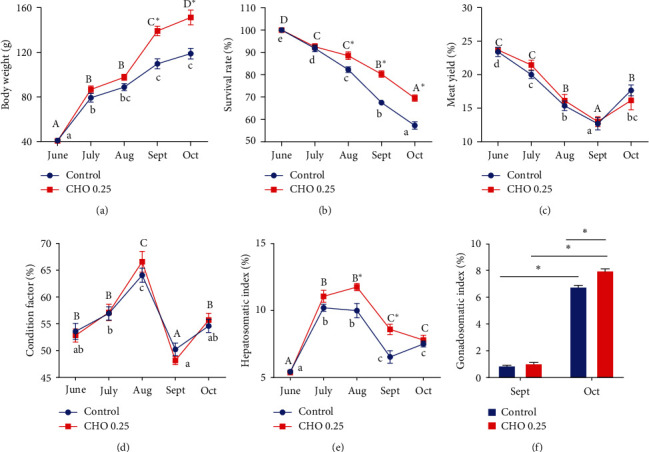
(a–f) Effects of supplement cholesterol and treatment time on growth performance of *Eriocheir sinensis*. Different letters indicate significant difference among different treatment time within the same cholesterol level (*P* < 0.05). The asterisk “ ^*∗*^” indicates significant difference between different cholesterol levels in the same treatment time (*P* < 0.05). Values are expressed as means ± SEM (*n* = 4).

As shown in Figure 1, at the same cholesterol level, the BW of crabs significantly increased with the increasing of treatment time, whereas SR results are reversed. At the same treatment time, the BW and SR of crabs supplemented with 0.27% cholesterol were significantly higher than that in control group (*P* < 0.05). Cholesterol level has no significant effect on MY and CF (*P* > 0.05). The HSI of crabs increased from June to August and then decreased at the same cholesterol level. The HSI of crabs fed with 0.27% cholesterol was significantly higher than that of the control group in August and September (*P* < 0.05). For GSI, there was no significant difference between the two groups in September, but the GSI of crabs increased significantly from September to October (*P* < 0.05). In October, the GSI of crabs in the 0.27% cholesterol group was significantly higher than that of the control group (*P* < 0.05).

### 3.2. Analysis of Antioxygenic and Immune Performance in Hepatopancreas

**Figure 2:**
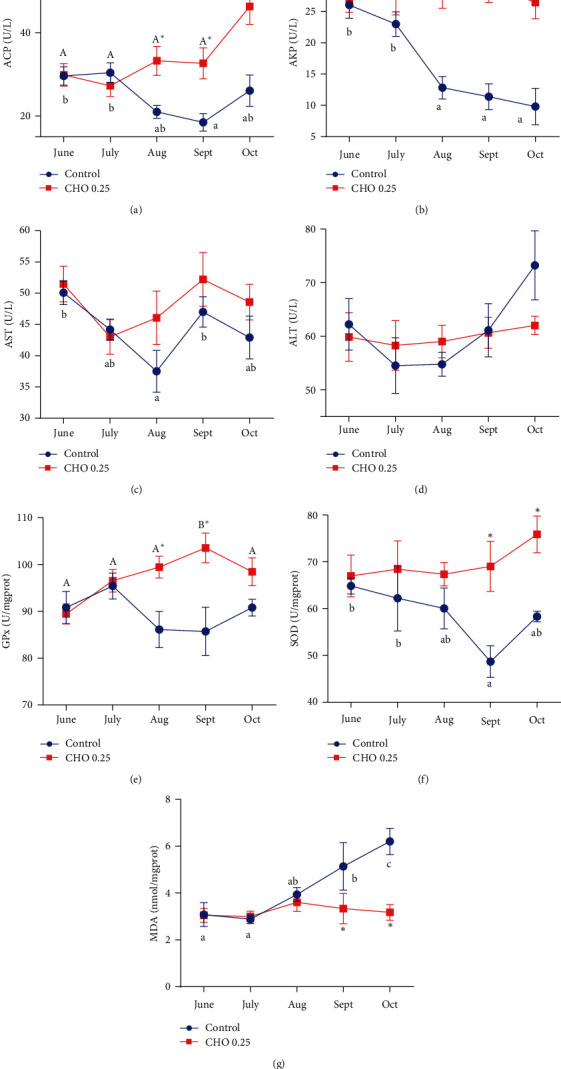
(a–g) Effects of supplement cholesterol and treatment time on antioxygenic and immune performance of *Eriocheir sinensis*. Different letters indicate significant difference among different treatment time within the same cholesterol level (*P* < 0.05). The asterisk “ ^*∗*^” indicates significant difference between different cholesterol levels in the same treatment time (*P* < 0.05). Values are expressed as means ± SEM (*n* = 4). ACP: acid phosphatase; AKP: alkaline phosphatase; AST: aspartate aminotransferase; ALT: alanine transaminase; GPx: glutathione peroxidase; SOD: superoxide dismutase; MDA: malondialdehyde.

The activities of ACP, AKP, GPx, and the content of MDA in hepatopancreas were significantly affected by the dietary cholesterol levels (*P* < 0.05) (Figure 2). Generally, 0.27% cholesterol can significantly increase the activity of ACP, AKP, and GPx and decrease the content of MDA (*P* < 0.05). However, the activities of AST and ALT were not influenced by the dietary cholesterol levels (*P* > 0.05). In addition, at the same cholesterol level, the treatment time had significant effects on ACP, AKP, AST, GPx, SOD, and MDA (*P* < 0.05). Generally speaking, the activities of ACP, AKP, AST, and SOD in cholesterol deficiency group showed a downward trend, while the content of MDA showed an upward trend.

### 3.3. The Analysis of Ovarian Histology

**Figure 3:**
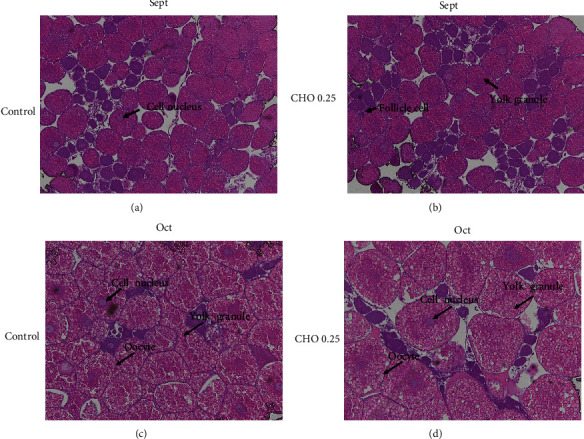
(a–d) Effects of supplement cholesterol and treatment time on ovarian histology of *Eriocheir sinensis (n* = 4). Photomicrographs (10x) and scale bar (100 *µ*m).

**Figure 4:**
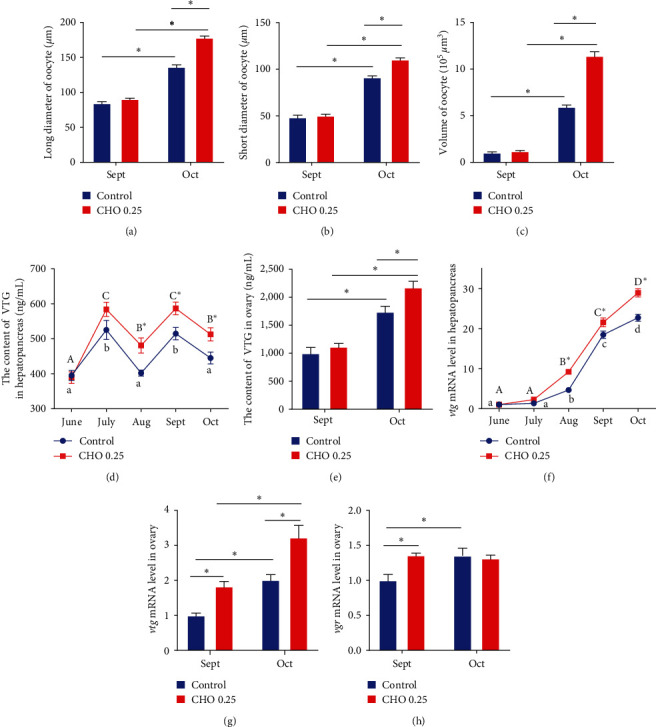
(a–h) Effects of supplement cholesterol and treatment time on the related parameters of oocytes and vitellogenin of *Eriocheir sinensis*. Different letters indicate significant difference among different treatment time within the same cholesterol level (*P* < 0.05). The asterisk “ ^*∗*^” indicates significant difference between different cholesterol levels in the same treatment time (*P* < 0.05). Values are expressed as means ± SEM (*n* = 4). VTG: vitellogenin; *vtg*: the mRNA expression level of vitellogenin receptor.

As shown in Figure 3, there was no significant difference in the number and volume of oocytes was observed between cholesterol supplemented group and the group without cholesterol in September. However, the volume of oocytes in the cholesterol-added group increased significantly, and yolk granules were more filling from September to October. The related parameters of oocytes were as shown in Figure 4(a)–4(c). Consistent with the results observed by sections, cholesterol levels had no significant effect on LO, SO, and VO in September, but in October, the above parameters in CHO 0.27% group were significantly higher than control group (*P* < 0.05).

### 3.4. The Analysis of VTG

The content of VTG in hepatopancreas and ovary was significantly affected by the treatment time (Figure 4(d)–4(h))(*P* < 0.05). The content of VTG in hepatopancreas first increased and then decreased from June to August and showed the same trend again from August to October; moreover, the cholesterol content in hepatopancreas in cholesterol treatment group was significantly higher than that in the control group from August (*P* < 0.05). For ovaries, the content of VTG increased significantly from September to October (*P* < 0.05), and compared with the control group, adding cholesterol significantly increased the content of VTG in ovaries (*P* < 0.05). According to the results of mRNA expression, the expression of *vtg* in hepatopancreas and ovary increased significantly with the increase of treatment time (*P* < 0.05), and cholesterol supplementation could significantly improve the expression of *vtg* (*P*  < 0.05). From September to October, the expression of *vgr* in the control group increased significantly (*P* < 0.05), but no significant difference was observed in the cholesterol treatment group. However, compared with the control group, the expression of *vgr* in cholesterol treatment group was significantly higher than that in the control group (*P* < 0.05).

### 3.5. The Change of Nutrient in Hepatopancreas

**Figure 5:**
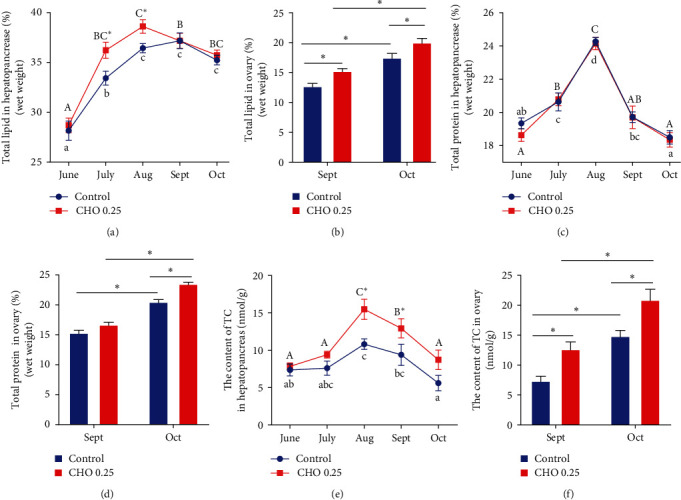
(a–f) Effects of supplement cholesterol and treatment time on nutrients in hepatopancreas and ovary of *Eriocheir sinensis*. Different letters indicate significant difference among different treatment time within the same cholesterol level (*P* < 0.05). The asterisk “ ^*∗*^” indicates significant difference between different cholesterol levels in the same treatment time (*P* < 0.05). Values are expressed as means ± SEM (*n* = 4). *vtg*: the mRNA expression level of vitellogenin; *vgr*: the mRNA expression level of vitellogenin receptor.

The content of lipid in hepatopancreas in cholesterol treatment group and control group increased first and then decreased with the increase of treatment time and reached the maximum at August and September, respectively (Figure 5). In addition, compared with the control group, the lipid content in hepatopancreas in cholesterol treatment group was significantly higher than that in July and Aug (*P* < 0.05). Similarly, the lipid content in ovaries also increased significantly with the increase of treatment time (*P* < 0.05). Moreover, cholesterol significantly increased the content of lipid in ovaries compared with the control group (*P* < 0.05). The content of protein in hepatopancreas and ovary was significantly affected by treatment time (*P* < 0.05). In the ovary, compared with the control group, cholesterol supplementation significantly increased the content of protein (*P* < 0.05). From June to October, the content of TC in hepatopancreas first increased and then decreased, and the content of TC in cholesterol added group was significantly higher than that in control groups in August and September (*P* < 0.05). In the ovary, the content of TC increased significantly from September to October (*P* < 0.05), and cholesterol supplementation could also significantly increase the content of TC (*P* < 0.05).

### 3.6. The Analysis of Gene Expression

**Figure 6:**
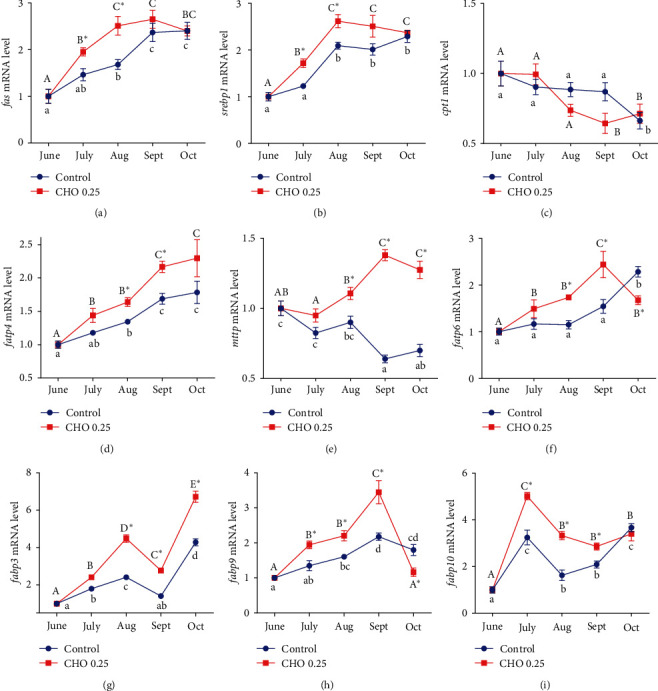
(a–i) Effects of supplement cholesterol and treatment time on the genes expression related to lipid metabolism. Different letters indicate significant difference among different treatment time within the same cholesterol level (*P* < 0.05). The asterisk “ ^*∗*^” indicates significant difference between different cholesterol levels in the same treatment time (*P* < 0.05). Data are expressed as means ± SEM (*n* = 4). *fas*: fatty acid synthase; *srebp1*: sterol regulatory element-binding protein 1; *cpt1*: carnitine palmitoyl transterase 1; *fatp4*: fatty acid transport protein 4; *mttp*: microsomal triglyceride transfer protein; *fatp6*: fatty acid transport protein 6; *fabp3*: fatty acid-binding protein 3; *fabp9*: fatty acid-binding protein 9; *fabp10*: fatty acid-binding protein 10.

According to the results of mRNA expression, dietary cholesterol level and treatment time significantly affected the gene expression of *mttp*, *fabp3*, *fabp9*, *fabp10*, *fatp6*, *srebp1*, *fas*, *cpt1*, and *fatp4 (P*  < 0.05) (Figure 6). Generally, the crab fed diet with 0.27% cholesterol showed higher gene expression of lipid transport-related genes (*mttp*, *fabp3*, *fabp9*, *fabp10*, *fatp6*) compared with the control group. The expression level of *fas* and *srebp1* in cholesterol-added group was significantly higher than control group in July and August. *Cpt1*, which is mainly affected by the treatment time, and its expression level has decreased as the treatment time increases. The expression level of *fatp4* was significantly influenced by dietary cholesterol level and treatment time. At the same cholesterol level, the expression level of *fatp4* showed an increasing trend with the increasing of treatment time. Under the same treatment time, the expression level of *fatp4* in the cholesterol- added group was significantly higher than that in the control group in August and September.

### 3.7. The Change of Steroid Hormones

**Figure 7:**
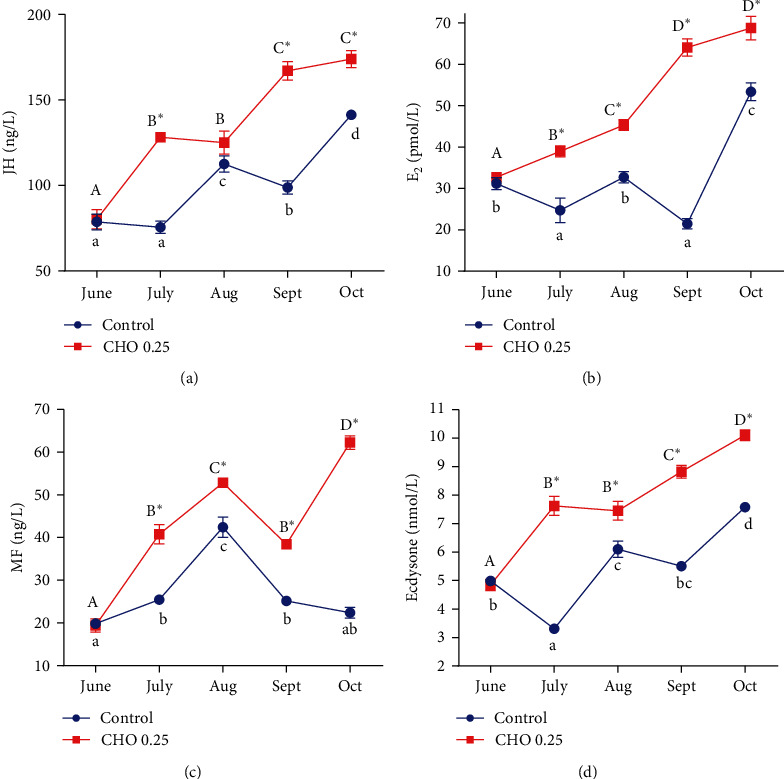
(a–d) Effects of supplement cholesterol and treatment time on hormone in the organism. Different letters indicate significant difference among different treatment time within the same cholesterol level (*P* < 0.05). The asterisk “ ^*∗*^” indicates significant difference between different cholesterol levels in the same treatment time (*P* < 0.05). Data are expressed as means ± SEM (*n* = 4). JH: juvenile hormone; E_2_: 17*β*-Estradiolum; MF: methyl farnesoate.

As shown in Figure 7, dietary cholesterol level and treatment time had significant effects on the level of JH, E_2_, MF, and ecdysone. Generally, in the same treatment time, the level of JH, E_2_, MF, and ecdysone in cholesterol-added group was significantly higher than that in the control group.

## 4. Discussion

Cholesterol is considered as an essential nutrient for the maturation and reproduction of shrimp and crabs [20, 29]. Previous study has reported that feeding cuttlefish (*Uroteuthis duvauceli*) can effectively promote gonadal maturation during the breeding process of *Litopenaeus vannamei* broodstock, further detection found that the cholesterol content of cuttlefish (*Uroteuthis duvauceli*) was as high as 16.1 mg/g [30]. However, with the increasing proportion of plant protein replacing fish meal in diet, the cholesterol content is often deficient. Our previous research has found that supplementing an appropriate amount of cholesterol in the diet can improve the growth performance of *Eriocheir sinensis* [9]. Similarly, in this study, we also found that long-term feeding of a diet with 0.27% cholesterol can significantly improve the BW, HSI, and GSI of *Eriocheir sinensis*. BW, HSI, and GSI are three important indexes in the process of broodstock breeding, which can directly determine the reproductive performance [10, 31]. In addition, cholesterol has no significant effect on the CF and MY of crabs, but the SR of cholesterol deficiency group is significantly reduced. This may be due to the long-term lack of cholesterol, which destroys the body's immune system and makes it more susceptible to infection by pathogenic bacterium [19]. To sum up, supplementing 0.27% cholesterol in the diet can improve the growth performance of *Eriocheir sinensis*.

The specific immunity of crustaceans is weak, and they mainly rely on nonspecific immunity to resist external infection [32]. Cholesterol is an indispensable part of maintaining cell membrane function, which is of great significance to the nonspecific immune function of the body [33, 34]. ACP and AKP are two important indexes to evaluate the nonspecific immunity of crustaceans [19, 35]. In this study, feeding diets lacking cholesterol for a long time will significantly reduce the levels of ACP and AKP in the organism, thus reducing the immunity of the body, which indicates that proper cholesterol can improve the immune performance of the body, and this result has also been reported in *Portunus trituberculatus* [19]. In addition, the antioxidant capacity of the body is also closely related to the health status of the body. Cholesterol may first act as an antioxidant to resist the infection of ROS, second, it may affect the synthesis and secretion of antioxidant enzymes by affecting the fusion of cell membranes, and last, cholesterol may also produce MF through mevalonate pathway to regulate the stress response of the body, so cholesterol can affect the antioxidant capacity of the body [36, 37]. In this experiment, the long-term lack of cholesterol will lead to a significant decrease in the activities of GPx and SOD, while the content of MDA will increase significantly. This shows that long-term lack of cholesterol will aggravate the degree of oxidative stress in the body, thus affecting the health status of the body.

VTG is a female-specific protein, which is the precursor of vitellin stored in oocytes. For crabs and shrimps, they rely entirely on the nutrition of vitellin to maintain their life within 48 hr after hatching, so vitellogenin plays a key role in reproductive success [24, 38]. Current research shows that the maturation of ovary is accompanied by the increase of oocyte volume, and the main reason for the increase of oocyte volume is the accumulation of vitellin, so vitellogenesis is considered as the central link of ovarian maturation [1, 17]. As a precursor of vitellin synthesis, VTG is mainly synthesized in adipocytes in hepatopancreas and ovary and then selectively absorbed by endocytosis mediated by VGR on the surface of oocytes [39]. The synthesis of VTG is regulated by endocrine hormones. It is found that JH, E_2_, MF, and ecdysone are several important hormones that regulate the synthesis of vitellogenin [15, 40, 41]. For example, JH can bind to Met in the nucleus and then regulate the transcription of *vtg* through transcription factor *kr-h1* [41]. At the same time, when oocytes recognize the signal of VTG in hemolymph, steroid hormones such as JH can promote the opening of follicular cells, so that VTG can reach the surface of oocytes and combine with VGR for absorption [17, 42]. In this study, we observed that in the early stage of ovarian development, there was no significant difference in the content of VTG between the cholesterol-treated group and the control group, but after entering the rapid gonadal development stage, the accumulation of VTG in the ovary of the cholesterol-added group increased significantly, which may be caused by the change of steroid hormones in the body [43, 44]. Therefore, this study speculates that the appropriate amount of cholesterol in the diet can increase the content of steroid hormones in the body, thus promoting the vitellogenesis and the transport of VTG and ultimately improving the ovarian maturity.

Vitellogenin is mainly synthesized from nutrients such as lipids, proteins, carbohydrates, and vitamins [17]. Therefore, the maturation of the ovaries cannot be separated from the accumulation of nutrients [4]. Previous studies have shown that lipids play an important role in the maturation of ovaries [28]. Before completing reproductive molting, the lipid content in the hepatopancreas increased rapidly, and HSI also increased, after entering the reproductive period, the ovaries will form another lipid metabolic center, during this period, HSI will begin to decline, while GSI gradually increases [45, 46]. This indicates that there is a large amount of nutrients was transferred during the process of ovary development, lipid and protein are the two most important substances, because they can provide sufficient nutrients for embryonic development and can participate in the synthesis of various hormones [47]. In this study, the contents of total lipid and total protein in hepatopancreas showed a trend of increasing at first and then decreasing, which indicated that in the early stage of ovarian development, the feed intake increased, and a large amount of nutrients were stored in hepatopancreas, but the ovary was not matured during this period, so only a small amount of nutrients were transported to the ovary. After the ovary enters the rapid development stage, a large amount of nutrients in hepatopancreas are transported to the ovary, which leads to the rapid increase of nutrients in the ovary in a short time, and this is consistent with the law of ovarian development in other decapod crustaceans [48–51]. However, interestingly, we found that supplementing cholesterol is more conducive to the transportation of nutrients. VTG is a carrier of nonpolar molecules, which is responsible for transferring nutrients from hepatopancreas to ovaries, and cholesterol is an important component of VTG [52]. Therefore, in this study, we further analyzed the influence of cholesterol in this process, and the results showed that the trend of cholesterol content in the body was highly consistent with fat and protein content. Compared with the control group, cholesterol significantly increased the content of VTG in hepatopancreas and ovary. Therefore, this study speculates that cholesterol can affect the transport of nutrients in hepatopancreas and ovary through VTG and then affect the process of ovarian maturation [53].

From the above results, we found that there is an obvious correlation between the changes of lipid content and cholesterol. So this experiment further explored the effect of cholesterol on lipid metabolism of *Eriocheir sinensis*. Unlike vertebrates, the digestion and absorption of lipid in *Eriocheir sinensis* mainly occur in hepatopancreas [54]. In this study, we found that the mRNA expression level of *srebp-1*, a transcription factor that activates the expression of key genes in downstream fatty acid synthesis, such as *fas*, which is a key rate-limiting enzyme for fatty acid synthesis, and its transcription level significantly increased after crabs were fed a cholesterol-containing diet, the results further confirm that cholesterol contributes to lipid accumulation in tissues, and similar results have been reported in mammals [11, 55–58]. In addition to self-synthesis, fatty acids in hepatopancreas can also be absorbed from the outside. However, hepatopancreatic cells need the assistance of FATP to absorb external fatty acids. FATP is a fatty acid transporter, which is mainly responsible for the transmembrane transport of fatty acids [59]. Overexpression of FATP can increase the level of fatty acid uptake by mouse myocardial cells by four times [60]. Fatty acids are highly hydrophobic, they cannot move freely in cells, so the lipid entering hepatopancreatic cells needs to be further transferred to the corresponding organelles by FABP [61]. For example, the lipid in hepatopancreatic cells can be further transported to lipid droplets for storage or transported to mitochondria for *β*-oxidation [62]. Cpt1 is a protein located in the outer membrane of mitochondria, which can promote fatty acids to enter the mitochondrial matrix and is the key rate-limiting enzyme for fatty acid *β*-oxidation [63]. In this study, the gene expression of *fatp* was significantly increased after adding proper cholesterol, thus enhancing the fat absorption ability of hepatopancreatic cells. In addition, compared with the control group, the expression of *fabp* also increased, which showed that cholesterol also enhanced the transport of fat in hepatopancreatic cells, but the expression of *cpt1* did not change significantly. Therefore, this study speculates that cholesterol did not affect the utilization of fatty acids by hepatopancreatic cells, and the fatty acids absorbed by hepatopancreatic cells may be transported to lipid droplets through FABP for subsequent ovarian development.

Hormones have been proved to play a key role in the reproductive and endocrine regulation of crustaceans [64]. Kazeto et al. [65] found that there was a significant correlation between the level of steroid hormones and the expression of *vtg* during ovarian maturation of *Eriocheir sinensis*. Cholesterol can participate in the synthesis of various hormones in the body [65], so this study examined the effects of cholesterol on several important reproductive hormones in the body [65–67]. E_2_, as the most active reproductive hormone in the body, plays an important role in regulating the growth and reproduction of animals [68]. There was some controversy about whether invertebrates can synthesize estrogen, but the existing evidence generally points to that invertebrates can also synthesize estrogen [69]. Pan et al. [44] found that estradiol can increase the mRNA expression of *vtg* in ovary through tissue culture in vitro. Ecdysone is a typical steroid hormone in crustaceans, which can be synthesized in the body with cholesterol as the substrate. Then it binds to ecdysone receptor and ultraspiracle protein, thus inducing the synthesis of VTG in hepatopancreas and finally promoting the maturation of oocytes [70]. The fertility of *Adelphocoris suturalis* decreased significantly after silencing the ecdysone receptor [71]. Both JH and MF belong to sesquiterpenoids, and JH can regulate a series of physiological processes such as growth and development, metamorphosis and reproduction of arthropods, which is one of the most important gonadotropins for insects and many crustaceans [72]. Moreover, JH can regulate the synthesis of VTG in fat body and can promote the uptake of VTG by oocytes by increasing the gap between follicular cells, thus promoting ovarian maturation [73]. MF is the direct precursor for the synthesis of JH, and the MF of crustaceans is very similar to JH of insects in physiological function and structure. MF is secreted by the mandibular organ of crustaceans [74–76]. Buchholz and Adelung [74] reported that compare the precocious crab with normal crab, the mandibular organ of precocious crab developed significantly faster. In this experiment, adding proper amount of cholesterol significantly improved the levels of the above hormones, which indicated that cholesterol could improve the reproductive performance of *Eriocheir sinensis* broodstock by increasing the levels of reproductive hormones in the body.

## 5. Conclusion

In conclusion, in the process of broodstock culture of *Eriocheir sinensis*, supplementation of appropriate amount of cholesterol can improve the growth performance and enhance the immune barrier, thus improving the health status. In addition, cholesterol also promotes the accumulation of nutrients in the hepatopancreas of crabs and enhances effectively the transport of nutrients from the hepatopancreas to the ovaries, thus promoting the vitellogenesis and the accumulation of vitellin. Meanwhile, as a substrate for the synthesis of various active substances, cholesterol also increases the contents of various reproductive hormones in the body, thereby promoting the maturation of the ovary and improving the reproductive performance.

## Figures and Tables

**Table 1:** The ingredients of experimental diets.

Ingredients (%)	Cholesterol levels (%)	
	0	0.25
Defatted fish meal^a^	18.50	18.50
Soybean meal	15.00	15.00
Rapeseed meal	2.50	2.50
Cottonseed meal	3.00	3.00
Peanut meal	28.50	28.50
*a*-Starch	19.00	19.00
EPA oil: DHA oil (1 : 1)^b^	1.20	1.20
Soybean oil	5.20	4.95
Carboxymethyl cellulose	1.00	1.00
Ca(H_2_PO_4_)_2_·H_2_O	2.20	2.20
Cholesterol (purity 99%)^c^	0.00	0.25
Lecithin	0.20	0.20
Zeolite	0.40	0.40
Premix^d^	1.00	1.00
Mixture^e^	2.30	2.30
Total	100.00	100.00
Proximate composition		
Crude protein	35.74	36.05
Crude lipid	7.98	7.82
Crude ash	6.89	7.02

^a^Fishmeal had been skimmed from 0.38% to 0.11% cholesterol. ^b^DHA oil and EPA oil (DHA content, 70% of oil; EPA content, 70% of oil) was purchased from Shanxi Pioneer Biotech Co., Ltd., Xian, Shanxi, China. ^c^Cholesterol (purity 99%) was purchased from Shanxi Pioneer Biotech Co., Ltd., Xian, Shanxi, China. ^d^Premix supplied the following minerals (g/kg) and vitamins (IU or mg/kg): CuSO_4_ · 5H_2_O, 2 g; FeSO_4_ · 7H_2_O, 25 g; ZnSO_4_ · 7H_2_O, 22 g; MnSO_4_ · 4H_2_O, 7 g; Na_2_SeO_3_, 0.04 g; KI, 0.026 g; CoCl_2_ · 6H_2_O, 0.1 g; vitamin A, 900,000 IU; vitamin D, 200,000 IU; vitamin E, 4500 mg; vitamin K3, 220 mg; vitamin B1, 320 mg; vitamin B2, 1090 mg; vitamin B5, 2000 mg; vitamin B6, 500 mg; vitamin B12, 1.6 mg; vitamin C, 10,000 mg; pantothenate, 1000 mg; folic acid, 165 mg; choline, 60,000 mg; biotin, 100 mg; and myoinositol 15,000 mg. ^e^Mixture includes the following ingredients (%): choline chloride 4.75%; antioxidants 1.72%; mildew-proof agent 2.35%; salt 22.06%; Lvkangyuan 59.30%, and biostimep 9.51%.

**Table 2:** Nucleotide sequences of the primers for real-time quantitative PCR.

Gene	Position	Primer sequence (5′−3′)	Length	Product size (bp)	Reference
*vtg*	Forward	AAGGTCCGCAGCAAGCAGAT	20	181	Lin et al. [28]
	Reverse	GGCGAGGCACGAGGTAGAAT	20		

*vgr*	Forward	GCAACGCCTTCCTTCTGGTA	20	193	Lei et al. [12]
	Reverse	GGCACGGTGTTCGCTATCAT	20		

*cpt1*	Forward	ATCTCCTCACCCGGCACTCT	21	183	Huang et al. [11, 16]
	Reverse	AGCAGGCAGTGGCTCAGTTTA	22		

*fas*	Forward	GTCCCTTCTTCTACGCCATCC	21	127	Lin et al. [13]
	Reverse	CGCTCTCCAGGTCAATCTTCAC	22		

*mttp*	Forward	TAGGACAAGCAGGACTTTCCTCA	23	138	Huang et al. [11, 16]
	Reverse	CCACATCCACAAACACATCAACA	23		

*Srebp1*	Forward	TCTTCACACCCTCTGGACGC	20	162	Huang et al. [11, 16]
	Reverse	CCAAGGTTGTAATGGCACGC	20		

*fabp3*	Forward	CCACCGAGGTCAAGTTCAAGC	21	195	KJ804230
	Reverse	TCACACCATCACACTCCGACAC	22		

*fatp6*	Forward	TGATGGGAAGGCAGGAATGG	20	119	Lin et al. [13]
	Reverse	TGCGGATGAAGCGAGGTACA	20		

*fabp9*	Forward	GGGCAACAAAATGACCCATAAG	22	108	GU568242
	Reverse	TGGCGAACACGCACAATCCT	20		

*fabp10*	Forward	TGATTGGCTCAGTGCTGTGGGT	22	115	HM459893
	Reverse	GGTGTTGGTGAAGTTCTTGTCGC	23		

*fatp4*	Forward	GACGGCAGACACGGAAAGAGA	22	101	Lin et al. [13]
	Reverse	CAGGTGGAGGCAAGCAAACTC	21		

*β-actin*	Forward	TCGTGCGAGACATCAAGGAAA	21	178	KM244725.1
	Reverse	AGGAAGGAAGGCTGGAAGAGTG	22		

*vtg*: vitellogenin; *vgr*: vitellogenin receptor; *cpt1*: carnitine palmitoyl transterase 1; *fas*: fatty acid synthase; *mttp*: microsomal triglyceride transfer protein; *srebp1*: sterol regulatory element-binding protein 1; *fabp3*: fatty acid-binding protein 3; *fatp6*: fatty acid transport protein 6; *fabp9*: fatty acid-binding protein 9; *fabp10*: fatty acid-binding protein 10; *fatp4*: fatty acid transport protein 4.

## Data Availability

Data will be made available on request.
